# Influence of Cooling Strategies on Surface Integrity After Milling of NiTi Alloy

**DOI:** 10.3390/ma18235472

**Published:** 2025-12-04

**Authors:** Małgorzata Kowalczyk

**Affiliations:** Cracow University of Technology, Faculty of Mechanical Engineering, Jana Pawła II 37 Avenue, 31-864 Krakow, Poland; malgorzata.kowalczyk@pk.edu.pl

**Keywords:** milling, NiTi alloy, surface roughness, TOPSIS method, Taguchi

## Abstract

Nickel–titanium (NiTi) alloys are extensively utilised in aerospace, biomedical, and precision engineering applications due to their distinctive functional properties, including superelasticity and the shape memory effect. However, their poor machinability and strong sensitivity to cutting conditions render it challenging to obtain surfaces with stable functional integrity. The present study investigates the impact of diverse cooling methodologies—namely dry machining, minimum quantity lubrication (MQL) and cryogenic cooling employing liquid nitrogen (LN2)—on the three-dimensional (3D) surface topography of NiTi alloy following milling. A comprehensive set of three-dimensional surface roughness parameters was employed to quantify the surface geometry and evaluate its potential functional performance. The findings indicated that both dry milling and MQL yielded significantly divergent surface parameters, suggesting unstable surface formation, which may potentially compromise component durability. MQL frequently resulted in topographies that were functionally detrimental and characterised by high parameter dispersion. In contrast, cryogenic cooling (LN2) resulted in the most homogeneous surface topography, as evidenced by the lowest dispersion of 3D roughness indicators. To strengthen the analysis, a Taguchi–TOPSIS multi-criteria optimisation was also performed on ten 3D surface parameters, enabling an integrated ranking of all machining trials. The optimisation process confirmed the superior performance of cryogenic machining, with LN2 conditions achieving the highest overall surface quality index.

## 1. Introduction

Nickel–titanium alloy (NiTi), widely known as Nitinol, belongs to a group of smart materials that exhibit the shape memory effect (SME) and superelasticity. Thanks to these unique properties, NiTi has found extensive applications in the medical industry (e.g., implants and stents), as well as in the aerospace and automotive engineering industries and in the manufacture of precision devices. Its exceptional mechanical behaviour is due to the alloy’s ability to undergo reversible martensitic transformation in response to changes in temperature or mechanical stress [1,2].

However, NiTi is also considered a difficult-to-machine material. Its high elasticity, low thermal conductivity, strong adhesion tendency and propensity for built-up edge formation, as well as rapid work hardening, lead to accelerated tool wear and deterioration of surface quality [3,4]. Furthermore, its high hardness and poor heat dissipation characteristics can lead to significant temperature increases in the cutting zone, potentially degrading the material structure and altering surface-layer properties [5,6]

For these reasons, selecting appropriate machining parameters and an effective cooling strategy is crucial for achieving high surface quality and extending tool life [7]. Although conventional cooling using metalworking fluids (e.g., oil–water emulsions) is commonplace, concerns about its environmental and health impacts have led to increased interest in more sustainable methods, such as minimum quantity lubrication (MQL) and cryogenic cooling using liquid nitrogen (LN2) [8,9]. The literature indicates that these sustainable strategies offer significant advantages. Cryogenic cooling (LN_2_) has proven particularly effective for difficult-to-machine alloys as it efficiently reduces temperature, minimises tool wear and limits the formation of built-up edges while maintaining a clean process [10,11,12]. Specific studies confirm this: Sun et al. [9] demonstrated that cryogenic cooling significantly reduces tool wear and improves surface finish when turning Hastelloy X compared to dry and MQL cutting. Wei et al. [3] reported that the application of cryogenic cooling during the machining of NiTi alloys leads to a noticeable reduction in cutting-zone temperature, which contributes to lower residual stresses and promotes the formation of a more homogeneous surface layer. Similarly, MQL has been shown to reduce cutting forces and improve surface quality [12]. Altas et al. [13] demonstrated that using MQL—particularly with the EG + 5%BX lubricant—significantly reduces cutting forces, limits tool wear, and improves surface quality during the milling of NiTi alloys, which the authors attribute to the favourable thermal conductivity and lubricating behaviour of this mixture. Zhao et al. [14], who investigated turning of NiTi under dry, flood and cryogenic LN_2_ conditions, similarly reported that both MQL and cryogenic cooling can reduce cutting forces and tool wear, although their effectiveness strongly depends on cutting speed and the ability of the lubricant to reach the cutting zone. Both studies consistently emphasise that proper delivery of coolant and lubricant decreases the temperature in the cutting zone and contributes to improved surface integrity.

In addition to the cooling strategy itself, existing studies emphasise that the resulting surface integrity of NiTi is strongly dependent on the interaction between the cutting parameters employed and the cooling–lubrication method applied. During dry machining, an increase in cutting speed usually results in a rapid accumulation of temperature, which intensifies adhesion, built-up edge formation and micro-tearing. This produces an irregular and highly dispersed surface topography [11]. Favourable effects on friction and cutting temperature have been reported for MQL-assisted cutting, although process stability strongly depends on the oil mist’s ability to penetrate the cutting zone. At higher feeds and speeds, inconsistent droplet delivery can result in local defects [8,11]. In contrast, cryogenic liquid nitrogen (LN_2_) cooling generally stabilises the machining process across a wide range of cutting speeds and feeds, effectively suppressing thermal–mechanical disturbances and reducing the formation of extreme surface features [7,11]. These findings suggest that when evaluating the expected surface integrity of NiTi alloy components, the selected cooling strategy must be considered jointly with the cutting speed and feed per tooth.

It is also important to note that many existing studies on NiTi machinability predominantly rely on two-dimensional (2D) profile parameters, such as *Ra* (average roughness). While these line-based metrics are useful, they fail to capture the full complexity and functional nature of the machined surface. A 2D profile only represents a single trace and can therefore overlook crucial anisotropic features, localised defects or the true nature of peaks and valleys, all of which are fundamental to functional performance [15,16]. Three-dimensional (3D) areal topography, as defined by parameters in ISO 25178 (e.g., *Sa*, *Sz*, *Sp*) [17], offers a more comprehensive and functionally relevant assessment of the surface’s geometric structure. This areal approach is essential for predicting properties such as friction, wear, fluid retention and biological integration (biocompatibility), which are often the primary functional requirements for NiTi components [18,19]. This view is consistent with broader findings in functional surface engineering, where 3D areal parameters and material ratio curves have been shown to be far more predictive of load-bearing behaviour and tribological performance than traditional 2D metrics [20].

Despite this growing body of research, a distinct research gap persists. Comprehensive studies directly comparing these cooling strategies (dry, MQL and LN_2_) under identical milling conditions are scarce. This is particularly true with regard to their influence on three-dimensional (3D) surface topography and its associated functional implications.

The surface integrity of NiTi after machining is of paramount importance. This holistic concept encompasses not only the geometric topography (the primary focus of this study), but also the underlying physical and metallurgical state of the surface layer [18,19,20,21]. This includes phenomena such as residual stresses, strain hardening and microcrack formation. Although these subsurface properties often require destructive or complex measurement techniques, the 3D surface topography itself can serve as a critical and sensitive indicator of machining process stability. High dispersion in 3D topography parameters often indicates an unstable process, which implies aggressive thermal and mechanical loading and suggests a high risk of compromised subsurface integrity. Even slight surface damage or unfavourable topography can reduce performance, particularly in medical and mechanical applications, where NiTi components must retain high fatigue resistance. Therefore, optimising cooling strategies is important for economic, functional, and material reasons [14,22,23,24].

This study presents the results of an experimental investigation into the influence of three cooling strategies—dry milling, minimum quantity lubrication (MQL) and cryogenic cooling (LN_2_)—on the surface integrity of NiTi alloy after milling. The experiments were conducted based on a Taguchi design to systematically evaluate variable cutting parameters. It is imperative to accentuate the fact that the present study has been meticulously designed to prioritise the three-dimensional (3D) surface topography as the principal metric of surface integrity. In view of the highly heterogeneous and locally disturbed nature of surfaces generated during NiTi milling, three-dimensional areal parameters provide the most reliable means of capturing spatial features that cannot be described using two-dimensional metrics alone. A number of aspects of surface integrity, including but not limited to residual stresses, microhardness and microstructural observations, require independent experimental procedures. It was therefore decided that these aspects would be intentionally excluded from the scope of this study. The present study focuses on a comprehensive set of 3D roughness and functional parameters, enabling detailed evaluation of the impact of cooling strategies on the formation of the surface layer.

## 2. Materials and Methods

**Figure 1:**
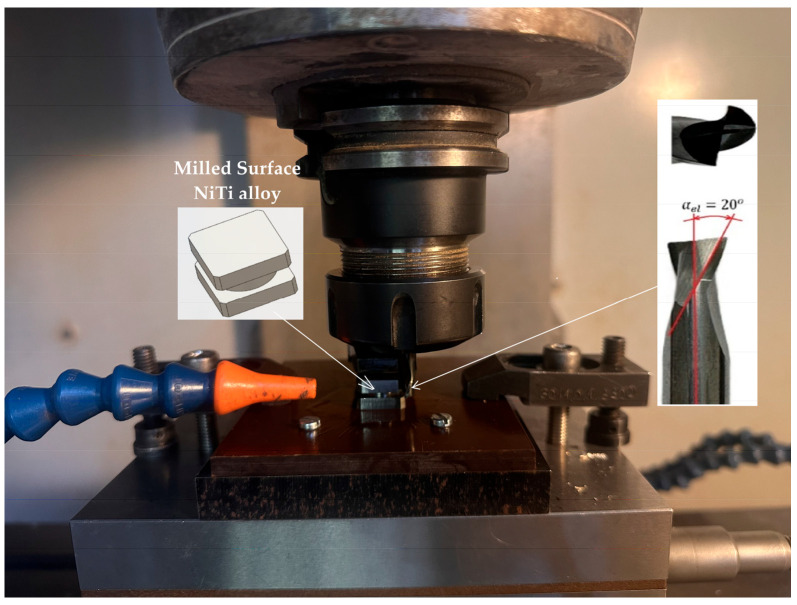
Experimental set-up for milling of NiTi.

The machining experiments were carried out on a three-axis CNC vertical machining centre (Haas VF-1) equipped with the Haas control system (Figure 1). The workpiece material used in the study was NiTi alloy exhibiting shape memory and superelastic behaviour, with an austenite finish temperature of A_f_ = 60 °C, obtained from Baoji Hanz Metal Material Co., Ltd. (Baoji, China). The experimental samples were prepared from the bar using wire electrical discharge machining (WEDM). This method was chosen to ensure high dimensional accuracy and prevent mechanical deformation, residual stresses or surface damage before milling. Each sample was rectangular in shape, measuring 10 mm by 4 mm. The 4 mm height corresponded directly to the axial depth of cut (*a*_p_ = 4 mm) used in the experiments.

Table 1 shows the chemical composition (wt. [%] and at. [%]) of NiTi. The composition was determined using EDS, where the Ti-Kα and Ni-Kα spectral lines were used for quantification; however, only elemental symbols (Ti, Ni) are reported here for clarity. The physical, thermal and mechanical properties of the materials of β-NiTi of the material are shown in Table 2, respectively.

**Figure 2:**
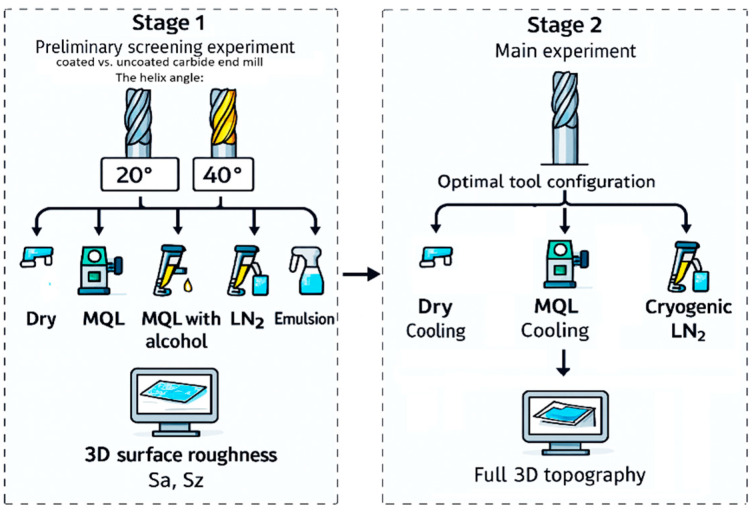
Schematic illustration of the experimental stages.

The research procedure involved two sequential experimental stages (Figure 2). The first, a screening-based experiment, was designed to identify the most favourable tool conditions and cooling media for NiTi milling. Only two surface roughness parameters (*Sa* and *Sz*) were measured in this preliminary experiment, as the purpose of this phase was not to perform a full evaluation of surface integrity, but rather to eliminate ineffective combinations of tool type and cooling strategy. A total of 20 machining trials were conducted. During these trials, the process parameters were held constant at a cutting speed (*v*_c_) of 55 m/min, a feed per tooth (*f*_z_) of 0.06 mm/tooth, an axial depth of cut (*a*_p_) of 4 mm, and a radial depth of cut (*a*_e_) of 0.4 mm. The investigation focused on three varied factors: the helix angle of the milling cutter (20° or 40°), the tool coating (uncoated vs. AlTiN-coated), and the type of cooling medium. The five tested media included a spirit-based coolant, kerosene, a conventional emulsion, minimum quantity lubrication (MQL), and cryogenic cooling using liquid nitrogen (LN_2_).

The results of this stage enabled the influence of the helix angle, coating, and coolant type on the basic geometric characteristics of the machined surface to be determined, thereby facilitating the rational selection of the most promising variants for the main part of the study. A brief analysis of the preliminary trials revealed that the 20° helix angle, AlTiN-coated tools and cryogenic cooling (LN_2_) produced the lowest *Sa* and *Sz* values consistently. Spirit- and kerosene-based cooling resulted in unstable surface formation, whereas emulsion and MQL produced intermediate but less consistent results. Based on these observations, the coated tool with a 20° helix angle and the three representative cooling strategies (dry, MQL and LN_2_) were selected for the main experiment.

The second stage, or main experiment, focused on comprehensively analysing the impact of cooling strategy and key machining parameters on the surface integrity of the NiTi alloy. The experimental design for this phase was highly structured: a Taguchi L9 orthogonal array was employed to investigate two machining parameters (Table 3), each varied across three levels: cutting speed (*v*_c_: 35, 55, and 75 m/min) and feed per tooth (*f*_z_: 0.02, 0.04, and 0.06 mm/tooth). Crucially, this entire L9 plan was then replicated for each of the three cooling strategies investigated: dry milling, MQL, and cryogenic (LN_2_). The delivery conditions for both cooling-lubrication strategies were standardised (Figure 1), with nozzles aligned toward the tool–chip–workpiece contact zone at comparable angles. A vegetable ester-based oil-mist unit supplied lubricant at a rate of 30 mL/h, atomised by compressed air at 6 bar. For cryogenic cooling, liquid nitrogen (LN_2_) was delivered through an insulated nozzle directed at the cutting zone The LN_2_ flow was maintained at 0.5–0.7 L/min to ensure a continuous cooling jet. This resulted in a robust dataset of 27 (9 × 3) total experimental runs, allowing for a direct comparison of the cooling strategies across an identical range of machining parameters. To ensure that the observed changes in surface topography primarily stemmed from the investigated factors (cooling, *v*_c_, and *f*_z_), the axial (*a*_p_) and radial (*a*_e_) depths of cut remained constant at 4 mm and 0.4 mm, respectively. All experiments in this stage were conducted using the tool variant identified in Stage 1 as producing the best surface finish; specifically, an AlTiN-coated solid carbide end mill with a 20° helix angle.

The Taguchi method was chosen as the primary framework for the main experiment. This statistical approach is specifically designed for process optimisation and robust design. One of the main advantages of this method is its efficiency. It utilises orthogonal arrays (OA), such as the L9 matrix used here. The L9 array enabled a systematic investigation of the effects of two machining parameters (*v*_c_ and *f*_z_) at three levels in just nine experimental runs. A critical component of this study was replicating this entire L9 matrix for each of the three cooling strategies (dry, MQL and LN_2_). This design, comprising 27 total runs (9 × 3), was ideally suited to applying Taguchi’s primary analytical tool: the signal-to-noise (S/N) ratio. To strengthen the methodological framework of the study, a Taguchi–TOPSIS multi-criteria optimisation was incorporated alongside the experimental analysis. Instead of relying on individual roughness metrics, the Taguchi–TOPSIS integration enabled a structured multi-parameter assessment of all 27 machining trials, allowing the overall surface integrity performance to be evaluated in a consistent and comparative manner.

As the primary objective was to identify a cooling strategy that yields the best average surface quality (‘the signal’) and exhibits the lowest variability (‘the noise’) in response to changes in *v*_c_ and *f*_z_, this method was ideally suited to determining the most robust and stable milling process.

The 3D geometric surface topography of the milled NiTi samples was investigated using a Taylor Hobson contact stylus profilometer (Taylor Hobson, Leicester, UK). The measurements were performed under controlled laboratory conditions (temperature: 20 ± 0.5 °C; relative humidity: 45–55%) using a calibrated Taylor Hobson roughness standard to verify the accuracy of the instrument prior to data acquisition. A constant stylus force of 0.75 mN was applied to prevent surface damage during scanning.

Three-dimensional measurements were conducted over a 1.0 × 1.0 mm area. A topography map was constructed from 100 parallel line profiles, corresponding to an inter-track spacing of 10 µm (raster step). Each profile consisted of 1000 registered points, corresponding to a sampling step of 1 µm along the line (Δ_x_). A stylus with a tip radius (*r*_tip_) of 2 µm was used for scanning, moving at a constant scanning speed (*v*_os_) of 1 mm/s.

The acquired raw data was subsequently imported into TalyMap Silver 5.0.3.5052 software for processing and visualisation. Standard pre-processing was applied, including noise and form removal and Gaussian filtering. To assess measurement uncertainty and natural process variability, each sample was measured three times. The reported values represent the arithmetic mean of these repeated measurements. All areal surface parameters were calculated in accordance with the ISO 25178 standard, and the averaged values were used in subsequent analyses.

## 3. Analysis of the Results

**Figure 3:**
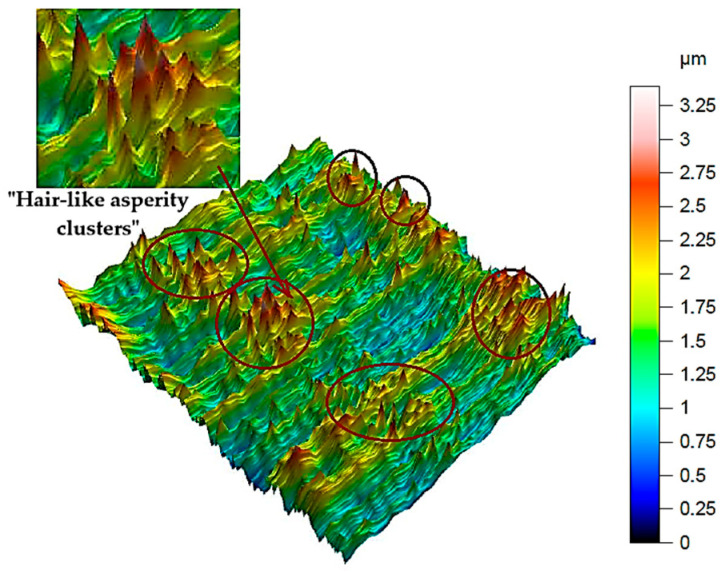
3D surface topography of the NiTi alloy after dry milling (*v_c_* = 35 m/min, *f*_z_ = 0.02 mm/tooth), showing local “hair-like asperities”.

A comprehensive evaluation of surface integrity after milling the NiTi alloy requires an analytical approach that extends beyond traditional 2D roughness parameters. Although 2D metrics such as *R*a or *R*z are widely used in machining studies, they provide only a line-based approximation of the surface profile and therefore do not sufficiently capture the spatial complexity of the surface generated during milling of shape-memory alloys. The NiTi alloy is particularly prone to producing heterogeneous, locally disturbed surface features resulting from its high elasticity, tendency to work-harden, and characteristic chip-tool interaction. As shown in Figure 3, the machined surface exhibits localised protrusions and depressions, as well as anisotropic ridge-like patterns, which arise from transient tool–material adhesion events, micro-tearing, and elastic recovery of the material. These irregularities are highly localised and non-periodic, making them undetectable or severely understated when using 2D parameters recorded along a single measurement trace.

For this reason, the analysis in the present work is based on a full set of 3D areal surface parameters, which enable a multidimensional and function-oriented characterisation of the surface topography. This includes amplitude parameters (e.g., *Sa*, *Sq*, *Sp*, *Sv*, *Sz*), parameters describing the height distribution (e.g., *Ssk*, *Sku*), and key functional metrics (e.g., *Smr*, *Smc*, *Sxp*). This combination provides complementary information related to the height distribution, peak and valley characteristics, material ratio, and load-bearing capacity of the surface. It also allows for distinguishing between surfaces that may show comparable average roughness values but fundamentally differ in their spatial structure, peak sharpness, or functional bearing behaviour.

Emphasising 3D parameters is essential, as the NiTi surface after milling is often dominated by directional patterns, micro-valleys, peak clusters, and localised surface disruptions, which cannot be reliably quantified by 2D analysis. The use of 3D topography therefore ensures a more realistic representation of the actual surface condition, enabling an accurate comparison of cooling and lubrication strategies, and providing insights relevant for both performance and fatigue-critical applications of NiTi components.

In accordance with this 3D-centric methodology, Table 4 presents the complete results of the experimental campaign.

This table summarises the average measured values of the key areal surface parameters for all 27 machining trials, which correspond to the L9 orthogonal array replicated across the three cooling strategies (dry, MQL and LN_2_). This dataset forms the basis for further analysis.

### 3.1. Amplitude Surface Parameters (Sa, Sq, Sp, Sv, Sz)

**Figure 4:**
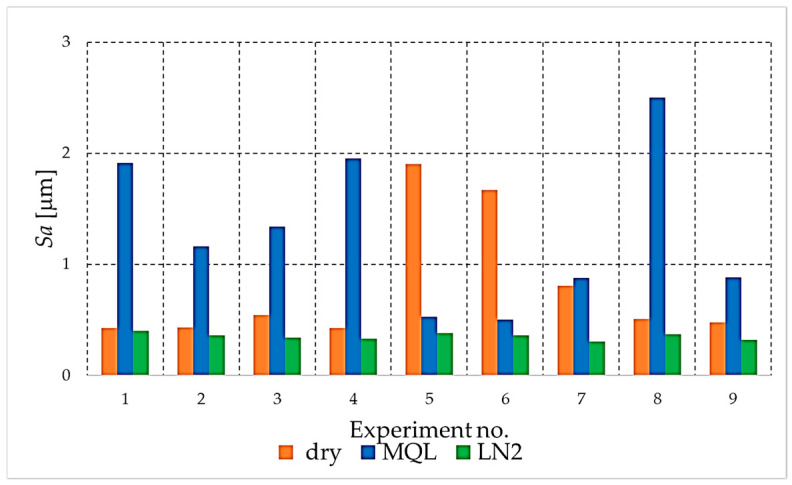
The comparison of the Sa parameter values for different cooling strategies.

**Figure 5:**
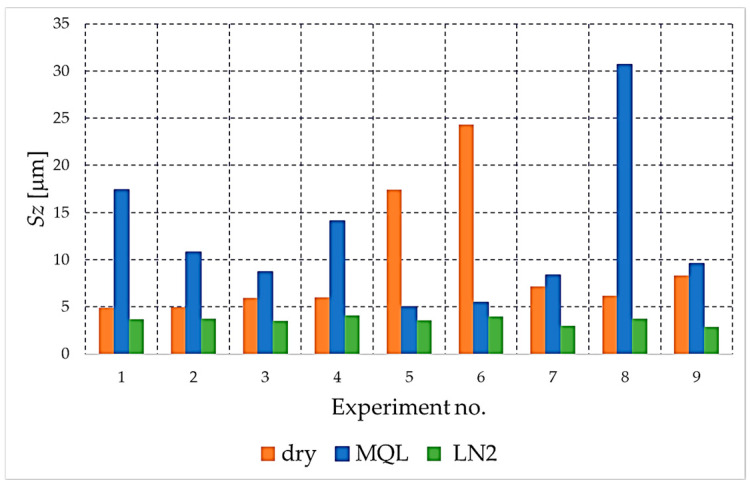
The comparison of the *Sz* parameter values for different cooling strategies.

**Figure 6:**
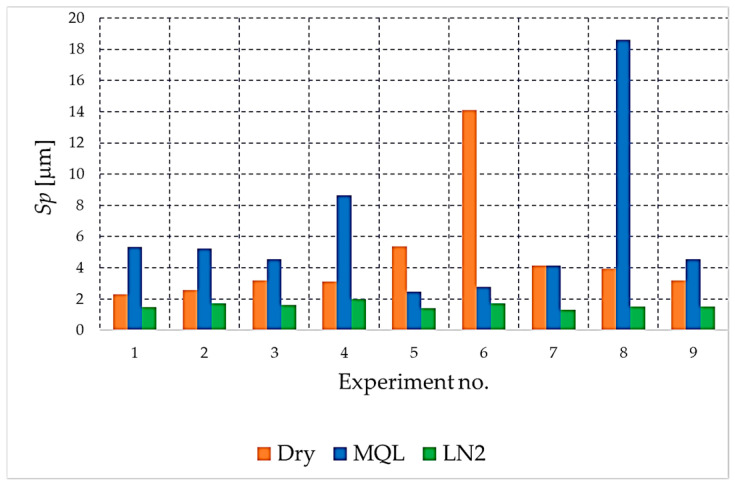
The comparison of the *Sp* parameter values for different cooling strategies.

**Figure 7:**
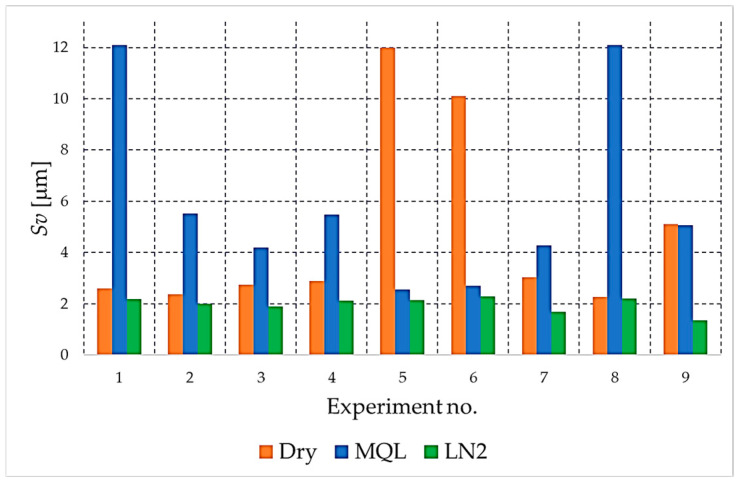
The comparison of the *Sv* parameter values for different cooling strategies.

**Figure 8:**
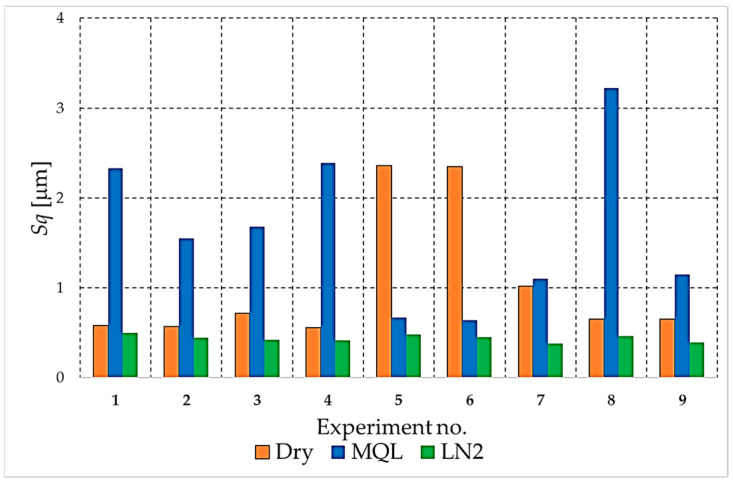
The comparison of the *Sq* parameter values for different cooling strategies.

The analysis begins with the amplitude parameters (e.g., *Sa*, *Sq*, *S*z, *Sp*, *Sv*), as these are fundamental indicators that directly quantify vertical deviation, overall height and the characteristics of peaks and valleys. Figure 4, Figure 5, Figure 6, Figure 7 and Figure 8 present the results for these parameters in the form of clustered bar charts. On each chart, the horizontal axis represents the nine experimental runs (Exp. No. 1–9) from the Taguchi L9 orthogonal array. Each run corresponds to a unique combination of cutting speed (*v_c_*) and feed per tooth (*f_z_*).

The three clustered bars at each experiment number show the measured value for the dry, MQL and LN_2_ strategies, respectively, performed at that specific parameter combination. This presentation format enables direct and detailed comparison of the influence of each cooling strategy on the outcome under identical machining conditions across the entire investigated area.

As demonstrated in Figure 4, the values of the arithmetic mean height parameter (*Sa*) are presented for the three cooling and lubrication conditions. The cryogenic liquid nitrogen (LN2) strategy consistently yielded the lowest *Sa* values across all cutting conditions, reflecting its ability to effectively suppress micro-irregularities and limit thermally induced deformation of the NiTi surface. Conversely, both MQL and particularly dry milling resulted in conspicuously elevated *Sa* values, suggesting increased peak formation, local material pull-out, and adhesion-related disturbances. The elevated levels of *Sa* value observed in dry conditions directly reflect the intensified thermal–mechanical loads, which promote surface instability and deterioration of the machined texture.

As demonstrated in Figure 5, the *Sz* parameter (maximum surface height) reached its highest values during both MQL and dry machining. These conditions promoted the formation of pronounced peak–valley amplitudes, reflecting unstable chip formation, adhesion–detachment events, and intensified thermal–mechanical interactions at the tool–workpiece interface. Such extreme surface features have been shown to have a detrimental effect on the functional performance of NiTi components, particularly in applications where fatigue or contact loading are of significance. Conversely, the utilisation of cryogenic liquid nitrogen (LN_2_) cooling resulted in a substantial suppression of the formation of these extreme asperities. This approach consistently yielded the lowest values for the surface roughness (*Sz*) parameter, thereby indicating a more stable and uniform material removal mechanism.

As demonstrated in Figure 6 and Figure 7 the analysis of the maximum peak height (*Sp*) and maximum valley depth (*Sv*) provides further insight into the formation of extreme surface features that may have a critical influence on the functional behaviour of the NiTi surface.

As demonstrated in Figure 6, the *Sp* parameter demonstrates variation across the cutting conditions that are the subject of this analysis. The highest *Sp* values were consistently observed under MQL and dry milling, indicating the formation of sharp, isolated peaks resulting from unstable chip–tool interactions, adhesion–deformation cycles, and local material pull-out. In particular, extreme peaks appeared in dry milling at higher feeds and cutting speeds, which confirms the detrimental effect of insufficient lubrication and elevated thermal loads.

Conversely, cryogenic cooling (LN_2_) yielded the lowest *Sp* values in nearly all experimental combinations. This outcome demonstrates the effectiveness of liquid nitrogen (LN_2_) in suppressing peak formation, likely due to reduced thermal softening of the material, more stable chip segmentation, and the mitigation of adhesion mechanisms at the tool–workpiece interface.

The *Sv* parameter (Figure 7) demonstrated a comparable tendency. The primary focus of the present study is the observation that large valley depths were recorded for dry milling, where insufficient cooling and high tool–chip temperatures promoted deep micro-tearing, surface plucking, and the formation of irregular depressions. MQL also produced pronounced valleys, although these were generally less extreme compared with dry conditions.

It was demonstrated that liquid nitrogen (LN_2_) cooling exhibited the best results. The *Sv* values remained consistently low, thereby confirming the hypothesis that cryogenic conditions effectively suppress the formation of deep valleys by minimising thermal–mechanical degradation mechanisms and stabilising material removal. When considering both *Sp* and *Sv* in unison, it is evident that cryogenic cooling provides the narrowest peak–valley range, which in turn translates into a more uniform and mechanically favourable surface. Conversely, MQL and dry milling demonstrate significantly greater *Sp*–*Sv* variability, resulting in surfaces characterised by extreme asperities and valleys. This is a feature that is detrimental to both fatigue resistance and contact mechanics.

As demonstrated in Figure 8, the root-mean-square roughness parameter (*Sq*) evidently differentiates the three cooling strategies. The lowest *Sq* values are consistently obtained for cryogenic LN_2_ cooling, thus confirming its ability to stabilise the cutting process and minimise the statistical dispersion of surface heights.

Conversely, the highest *Sq* values are predominantly associated with the MQL strategy, particularly in experiments 1 and 8, where MQL produces significantly larger *Sq* than both dry and LN_2_ conditions. This finding suggests that, under specific combinations of cutting speed and feed, the presence of oil mist may not guarantee a reliable lubrication and cooling effect. This, in turn, can result in substantial surface height variations and the occurrence of severe topographical disturbances in specific localised areas.

Furthermore, dry machining has been shown to generate elevated *Sq* values, particularly in experiments 5 and 6. In the absence of coolant, significant height variability is observed, driven by thermal softening, adhesion and micro-tearing of the NiTi surface. However, in several tests, the *Sq* values for dry cutting remain lower than those measured under MQL, which highlights the strong parameter sensitivity and instability of the MQL process. The *Sq* analysis demonstrates a clear ranking of the applied strategies. LN_2_ provides the most homogeneous and stable surface, MQL often yields the largest *Sq* values and exhibits the greatest variability, and dry machining generally performs worse than LN_2_ but, in some cases, slightly better than MQL.

### 3.2. Height Distribution Parameters: Ssk and Sku

A thorough evaluation of the surface topography resulting from the milling of the NiTi alloy necessitates not only the consideration of amplitude parameters but also the utilisation of descriptors that effectively characterise the height distribution of the surface. In this context, skewness (*Ssk*) and kurtosis (*Sku*) provide essential information about the asymmetry and sharpness of the surface profile. Negative *Ssk* values are indicative of valley-dominated (left-skewed) surfaces, whereas positive values reflect a predominance of peaks (right-skewed distributions). The *Sku* parameter, conversely, describes the sharpness of topographical features: values close to 3 correspond to a mesokurtic, near-Gaussian distribution, while higher *Sku* values indicate the presence of sharp peaks or deep valleys, which are typical of a leptokurtic surface. It is therefore concluded that these parameters offer a functional interpretation of the machined surface layer, thus supplementing the information derived from amplitude measures.

**Figure 9:**
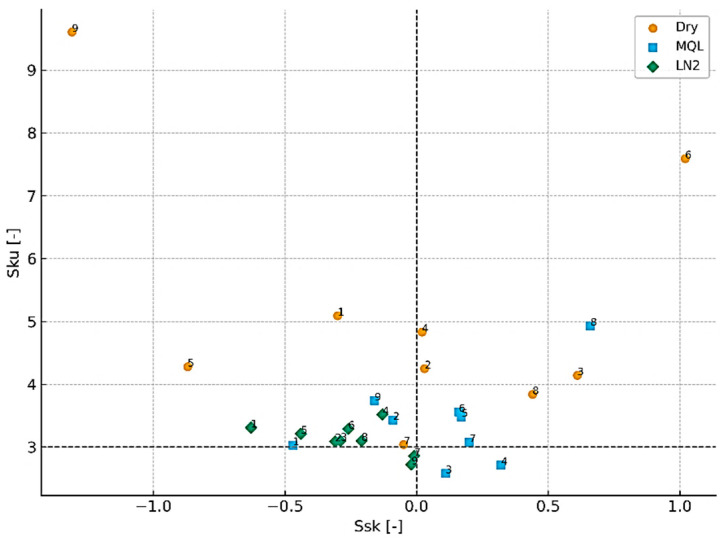
The distributions of *Ssk* and *Sku* under different cooling conditions.

Figure 9 presents the relationship between *Ssk* and *Sku* for all machining conditions that were tested. The findings unequivocally demonstrate that dry machining yields the most unstable and defect-prone surface topography. The skewness values range extensively, from −0.30 to +1.02, indicating that both valley-dominated and strongly peak-dominated profiles emerge contingent on the cutting parameters. The highest positive skewness, recorded for sample 12 (*Ssk* = +0.61), reflects sharp protrusions formed as a result of adhesion, micro-tearing and thermal softening. Concurrently, the kurtosis values associated with dry machining attain remarkably elevated levels (*Sku* up to 9.61), thereby substantiating the existence of narrow peaks and profound valleys, thus unveiling a conspicuously leptokurtic distribution. Such a surface morphology is indicative of unstable thermomechanical conditions in the cutting zone and correlates with the deterioration of the integrity of the machined layer.

In comparison, the MQL strategy leads to a more regular height distribution, although the surface remains susceptible to the formation of sharp peaks. The skewness values for this group range between −0.47 and +0.66, with the highest positive skewness and kurtosis observed for sample 26 (*Ssk* = +0.66, *Sku* = 4.93), indicating distinct protrusions despite lubrication. The *Sku* values for MQL (2.58–4.93) indicate that, while oil-mist lubrication mitigates certain deleterious effects associated with dry machining, it does not entirely suppress the thermomechanical irregularities that are characteristic of NiTi.

The most favourable and stable surface morphology is obtained under cryogenic liquid nitrogen (LN_2_) cooling conditions. The skewness values remain consistently negative or close to zero (from −0.63 to −0.01), indicating surfaces dominated by shallow valleys or nearly symmetric height distributions. Furthermore, kurtosis values remain close to the Gaussian reference (*Sku* ≈ 2.72–3.52), suggesting the absence of extreme spikes or deep material pull-outs. These results provide confirmation of the stabilising effect of LN_2_ cooling, which has been demonstrated to be effective in reducing adhesion, thermal accumulation and micro-damage in the surface layer.

The practical significance of analysing *Ssk* and *Sku* becomes particularly evident when comparing surfaces with similar *S*a values but fundamentally different structural characteristics. For instance, Tests 1 (LN_2_) and 11 (dry) demonstrate almost identical *Sa* values (0.40 µm vs. 0.43 µm), which may indicate comparable surface quality. However, analysis of their height-distribution parameters reveals entirely different topographies: the cryogenically machined surface shows *Ssk* = −0.63 and *Sku* = 3.31, indicating a stable, valley-oriented surface with no extreme features, while the dry-machined surface (*Ssk* = +0.03, *Sku* = 4.25) displays a peak-dominated profile with sharper features and a higher probability of micro-defects. A similar situation is observed for Test 17 (dry) and Test 24 (MQL), both of which are characterised by identical *Sa* values (0.50 µm). Notwithstanding this finding, the dry-machined surface demonstrates considerably higher skewness and kurtosis (*Ssk* = +0.44, *Sku* = 3.84) in comparison to the MQL surface (*Ssk* = +0.16, *Sku* = 3.56). This outcome serves to reinforce the conclusion that amplitude-based indicators are inadequate for distinguishing the functional quality of the surfaces.

These comparisons evidently demonstrate that two surfaces may possess the same average roughness value but differ substantially in their structural and functional characteristics. It is therefore vital to consider height-distribution parameters such as *Ssk* and *Sku* when conducting a comprehensive evaluation of surface integrity subsequent to milling the NiTi alloy. This enables more precise differentiation between surfaces that would otherwise appear similar when assessed solely on the basis of *Sa*.

### 3.3. Functional Surface Parameters: Smr, Smc and Sxp

Functional parameters of surface topography—namely the material ratio *Smr*, the core roughness depth *Smc*, and the extreme peak height *Sxp*—provide an essential complement to amplitude and height-distribution metrics, as they enable a functional, tribology-oriented interpretation of the machined NiTi surfaces. These parameters are strongly associated with the load-bearing capability of the surface, the formation and retention of lubricating films, and the susceptibility to local stress concentration, which is particularly relevant for fatigue-sensitive applications of NiTi alloys.

The material ratio (*Smr)* quantifies the proportion of material present at a defined cutting depth. A high *Smr* indicates that the surface contains a broad plateau region capable of distributing mechanical loads and reducing localised stresses. In contrast, low Smr values signify limited bearing support and a predominance of deep valleys, which may compromise wear resistance and coating adhesion. In the present study, cryogenic machining (LN_2_) consistently produced moderate to high *Smr* values (e.g., 11.4% in test 9 and 21.5% in test 7), reflecting uniform material distribution typical for stable cutting conditions. Conversely, dry machining frequently yielded extremely low Smr values, reaching as little as 0.038% (test 16), indicating a highly irregular structure dominated by deep depressions. Under MQL, *Smr* fluctuated considerably, with both elevated values (e.g., 2.71% in test 25) and very low values (0.12% in test 26), confirming the unstable character of this lubrication strategy.

The core material height (*Smc*) describes the thickness of the load-bearing core at a given material ratio. A lower *Smc* is generally associated with surfaces that retain lubrication efficiently and exhibit favourable tribological behaviour. Cryogenic cooling produced nearly uniform and low *Smc* values (0.75–1.47 µm), indicating stable core geometry and limited variability in peak-to-valley transitions. In contrast, dry milling resulted in markedly higher and more variable *Smc* values (e.g., 2.89 µm in test 16 and 11.5 µm in test 15). These elevated values reveal the presence of abrupt changes in topography and thicker load-bearing cores, suggesting a surface prone to mechanical instability and increased frictional interaction. The MQL strategy again demonstrated inconsistent behaviour, with Smc values ranging from moderate (1.20 µm in test 24) to very high (14.8 µm in test 26), reflecting heterogeneous lubrication and non-uniform chip evacuation.

The extreme peak height (*Sxp*) provides additional insight into the presence of isolated asperities capable of generating high local contact stresses. Low *Sxp* values obtained for cryogenic cooling (0.75–1.19 µm) confirm the absence of outliers and sharp peaks, which aligns with the nearly symmetric height distribution observed in *Ssk*–*Sku* analysis. Surfaces produced by dry milling exhibited substantially higher *Sxp*, particularly in tests affected by thermal instability (e.g., 2.29 µm in test 12 and 3.95 µm in test 15). Such elevated values are characteristic of uncontrolled material tearing, adherence–debonding events, or micro-chipping of the cutting edge. MQL conditions again produced the highest and most irregular *Sxp* values, reaching 5.46 µm in test 26, which reflects the formation of singular, unstable asperities associated with insufficient or uneven lubrication.

Overall, the collective interpretation of *Smr*, *Smc* and *Sxp* clearly indicates that cryogenic machining (LN_2_) provides the most favourable functional surface characteristics, delivering uniformly low peak heights, stable load-bearing core geometry and material ratios indicative of reliable tribological performance. Dry machining, on the other hand, introduces substantial surface irregularities, deep valleys and isolated extreme peaks, all of which diminish load-bearing capacity. MQL, despite its potential to reduce friction, demonstrated pronounced instability, with functional parameters showing the widest variability among all strategies.

The integration of functional surface parameters thus confirms that amplitude-based metrics alone (e.g., *Sa*, *Sq*) are insufficient to fully describe the behaviour of NiTi surfaces. Smr, Smc and Sxp reveal deeper topographical features that are crucial for understanding wear mechanisms, lubrication retention and the long-term durability of machined NiTi components.

### 3.4. Comparative 3D and Abbott–Firestone Analysis (LN_2_/Dry/MQL)

A complementary interpretation of the surface integrity after milling the NiTi alloy can be obtained by jointly analysing the three-dimensional topography and the corresponding Abbott–Firestone curves. This combination facilitates the quantification of both height distribution and amplitude variations, in addition to the evaluation of the bearing and functional behaviour of the real surface. This is of particular importance for components that are sensitive to fatigue or subject to tribological loading. As illustrated in Table 5, representative results are presented for cryogenic cooling (test 7), dry milling (test 15) and MQL oil-mist lubrication (test 26). These results demonstrate the characteristic effects of each cooling strategy on the spatial structure of the machined surface.

The surface produced under cryogenic liquid nitrogen cooling (test 7) displays the most favourable and uniform topography. The 3D map reveals shallow, evenly distributed asperities with no distinct outliers, while the Abbott curve shows a smooth progression and balanced material distribution. This behaviour is confirmed by the low amplitude parameters (*Sa* = 0.31 µm, *Sq* = 0.38 µm) and height distribution metrics close to the ideal mesokurtic profile (*Ssk* = −0.01, *Sku* = 2.86). The functional parameters further emphasise this advantage: the bearing ratio reaches *Smr* = 21.5%, while *Smc* = 0.83 µm and *Sxp* = 0.75 µm indicate minimal extreme peaks. Such a combination reflects a surface with high load-bearing capacity, stable tribological behaviour and a reduced tendency to form localised stress raisers. The cryogenically cooled surface is characterised by a number of features that render it particularly well-suited to applications requiring long-term durability, uniform contact conditions, and resistance to fretting or fatigue.

A contrasting surface structure is observed in dry machining (test 15), where the topography becomes markedly more irregular. The 3D map reveals the presence of sharp ridges, deep valleys and localised defects, which are attributed to thermal softening, adhesion–debris interactions and unstable chip formation. This is evidenced by the substantial asymmetry (*Ssk* = 1.02) and the remarkably elevated kurtosis (*Sku* = 7.59), suggesting the existence of conspicuous peak clusters and precipitous height transitions. The Abbott–Firestone curve steepens rapidly in the material core and exhibits an extended tail, which is consistent with the extremely large height range (*Sz* = 24.3 µm). The bearing ratio is found to be negligible (*Smr* = 0.05%), and both *Smc* (11.5 µm) and *Sxp* (3.95 µm) reach values that are indicative of highly defective surfaces. Such surface morphology would be disadvantageous in terms of cyclic loading, as the deep valleys may act as crack initiators, while the sharp peaks can undergo rapid abrasion or micro-fracture during service.

The MQL (oil-mist) strategy (test 26) resulted in the most severe surface degradation, exhibiting the highest amplitude parameters of all three conditions (*Sa* = 2.5 µm; *Sz* = 30.7 µm). The 3D map is characterised by the presence of notably elevated, solitary peaks (as evidenced by *Sp* = 18.16 µm) and substantial valleys. The topography is reflected in the height distribution, which is positively skewed (*Ssk* = 0.66) and leptokurtic (*Sku* = 4.93), indicating a “spiky” surface. The Abbott-Firestone curve corroborates this finding, demonstrating a precipitous profile with an extended tail, analogous to the dry condition. From a functional perspective, the surface in question is characterised by its substandard performance, as evidenced by the negligible bearing ratio (*Smr* = 0.05%) and the elevated core material height (*Smc* = 14.8 µm). The maximum peak height (*Ssp* = 5.46 µm) is also the most pronounced in the study, suggesting a surface dominated by singular asperities that would act as severe stress concentrators. This morphology is indicative of an unstable process, where the MQL was insufficient to prevent localised adhesion and material tearing.

As demonstrated in Table 5, cryogenic cooling has been shown to be a significantly more effective method of producing surfaces that are both functional and structurally advantageous. The process of dry milling has been shown to result in surfaces that are characterised by severe topographical disturbances. Conversely, the MQL method has been observed to generate inconsistent and peak-dominated structures, which are attributed to the unstable transport of lubricant. Conversely, LN_2_ produces a surface that is characterised by its smooth, symmetrical and mechanically robust nature, as indicated by its low extreme features and a favourable load-bearing profile.

It is important to note that the substantial differences observed in the 3D maps and Abbott–Firestone curves would not be detectable using mean parameters such as Sa alone. This finding underscores the imperative for conducting a comprehensive three-dimensional (3D) functional analysis when evaluating surface integrity, particularly in the context of advanced materials such as NiTi, where surface quality directly correlates with fatigue resistance, tribological behaviour and long-term durability.

## 4. Multi-Criteria Optimisation of Surface Integrity (Taguchi-TOPSIS Method)

The analysis of individual SGP parameters in the preceding subsections revealed complex and often competing trends. For instance, MQL conditions might sporadically improve one parameter while drastically degrading another. To identify the machining conditions that provide the best overall compromise—i.e., a surface that is simultaneously smooth (low *Sa*), defect-free (low *Sxp*), symmetrical (*Ssk* ≈ 0), and functional (high *Smr*)—a multi-criteria optimisation method is necessary.

In this work, a hybrid strategy combining the Taguchi design of experiments with the TOPSIS (Technique for Order Preference by Similarity to Ideal Solution) multi-criteria decision-making method was employed [24,26].

The first step was to translate the raw SGP data (from Table 4) into a common, logarithmic Signal-to-Noise (S/N) ratio scale. This transformation is essential as it allows for the comparison of different parameters, each with a distinct optimisation goal.

For all 27 trials, 10 different S/N ratios were calculated, according to the following criteria:

Smaller-is-Better (SB): Parameters: *Sa*, *Sq*, *Sp*, *Sv*, *Sz*, *Smc*, and *Sxp* (lower amplitudes and smaller peaks/valleys are desired).

Larger-is-Better (LB): Parameter: *Smr* (a higher material ratio improves load-bearing capacity).

Nominal-is-Best (NB): Parameters: *Ssk* (Target = 0, for symmetry) and *Sku* (Target = 3, for a mesokurtic distribution).

This transformation resulted in an S/N Decision Matrix (27 experiments × 10 criteria), in which a higher S/N value always represented a more desirable (closer to ideal) outcome.

The second step was to normalise and weight the data (TOPSIS Preparation). The Taguchi method itself only optimises one criterion at a time. To solve this, the TOPSIS method was used to synthesise all 10 S/N ratios into a single global index. The entire S/N transformation and TOPSIS algorithm were implemented using custom scripts written in MATLAB R2023a.

Normalisation: The S/N matrix (27 × 10) was first normalised using vector normalisation. This eliminated scale differences between the various S/N ratios.

Weighting: Each criterion (column) was assigned a weight (wj) according to its functional relevance for NiTi: Amplitude Parameters (*Sa*, *Sq*, *Sp*, *Sv*, *Sz*): Total Weight = 0.5; Distribution Parameters (*Ssk*, *Sku*): Total Weight = 0.2; Functional Parameters (*Smr*, *Smc*, *Sxp*): Total Weight = 0.3. The weighting scheme employed in the TOPSIS analysis was established on the basis of the functional relevance of each parameter group for surface integrity assessment in NiTi machining. The parameters of amplitude (50%) were assigned the highest level of importance, as they most directly reflect surface defects, thermal–mechanical instability and fatigue-related performance. The functional parameters (30%) were weighted according to their strong relevance for load-bearing capacity, lubrication retention and wear resistance (*Smr*, *Smc*, *Sxp*). The height-distribution parameters (20%) were assigned a complementary role, as they describe symmetry and sharpness of surface features (*Ssk*, *Sku*), yet play a secondary function in determining overall surface durability. This hierarchical structure is consistent with the trends reported in the surface-texture literature, where amplitude parameters are recognised as the most widely used and the most functionally significant descriptors of surface topography. Distribution-related and functional parameters provide supporting but still essential information. These conclusions are in alignment with the comprehensive review of surface-texture parameter relevance presented by Pawlus et al. [25].

Following the weighting, the core TOPSIS procedure was executed. This method identifies the optimal trial by calculating its relative proximity to an Ideal Best Solution (A+) and an Ideal Worst Solution (A−). The A+ vector was constructed by identifying the maximum (best) weighted S/N value from all 27 trials for each of the 10 parameters, while the (A−) vector was constructed from the minimum (worst) values.

Euclidean distances were then calculated for each of the 27 trials to both the ideal best (Di+) and the ideal worst (Di−). These two distances were synthesised into a single, comprehensive metric: the Closeness Coefficient (Ci), calculated as:

(1) $$C^{i}=D_{i}^{-}/(D_{i}^{+}+D_{i}^{-})$$

A *C*_i_ value approaching 1 signifies a “globally optimal” solution, one that is very close to the ideal best and far from the ideal worst [27,28].

The application of this procedure yielded a definitive ranking of all 27 machining conditions. The results of the analysis (presented in Table 6) provide unambiguous conclusions.

The cryogenic cooling (LN_2_) strategy demonstrated total dominance, occupying all top nine positions in the ranking. This indicates that LN_2_ produces a more consistent and robust surface, regardless of the specific *v_c_* or *f_z_* parameters used. The global optimum (Rank 1) was identified as Test no. 7 (LN_2_, *v_c_* = 75 m/min, *f_z_* = 0.02 mm/tooth), which achieved a *C_i_* score of 0.963. This confirms the qualitative analysis, as this test also exhibited a near-perfect symmetrical and mesokurtic distribution.

In stark contrast, the MQL strategy performed the poorest, occupying 7 of the 9 lowest-ranked positions. The worst-performing condition (Rank 27) was identified as Test no. 26 (MQL, *v_c_* = 75 m/min, *f_z_* = 0.04 mm/tooth), with a *C_i_* score of 0.081, correlating directly with the extremely poor functional parameters observed previously. The hybrid Taguchi-TOPSIS approach thus provides definitive proof that relying solely on *Sa* or *Sq* is insufficient. Only this holistic, multi-criteria assessment could confirm that cryogenic cooling is the only strategy that delivers a stable, robust, and functionally reliable surface integrity for NiTi machining.

## 5. Conclusions

This study provides a comprehensive and multi-level evaluation of the surface integrity of NiTi alloy after milling under three distinct cooling–lubrication strategies: cryogenic cooling with liquid nitrogen (LN_2_), dry cutting, and Minimum Quantity Lubrication (MQL). The integration of full three-dimensional surface metrology, height-distribution parameters, functional Abbott–Firestone analysis, and a multi-criteria optimisation approach based on the TOPSIS method demonstrates the inadequacy of traditional two-dimensional roughness indicators for assessing the spatial complexity and anisotropic nature of NiTi surfaces after machining. The 3D amplitude parameters revealed pronounced differences between the examined strategies, with cryogenic cooling consistently producing the lowest values of *Sa*, *Sq*, *Sz*, *Sp* and *Sv*. This finding indicates a highly uniform surface with minimal micro-defects. This trend was confirmed by height-distribution descriptors: The use of liquid nitrogen (LN_2_) machining resulted in surfaces exhibiting near-symmetrical and mesokurtic distributions. Conversely, dry and MQL conditions led to surfaces with strongly skewed and leptokurtic topographies, characterised by peak clustering, micro-tearing, and unstable chip formation. The investigation revealed that functional parameters, including *Smr*, *Smc* and *Sxp*, further demonstrated that cryogenic environments lead to surfaces with enhanced bearing capacity, controlled core depth and reduced risk of stress concentration. These properties are essential for the performance of fatigue-sensitive and tribologically loaded NiTi components.

It is important to note that the discrepancies observed between these surface states would not be identifiable based solely on average roughness parameters such as *Sa*. This underlines the necessity of employing full 3D functional characterisation in the evaluation of machined NiTi surfaces.

The study introduces a novel methodological framework that combines full 3D surface analysis, height-distribution parameters, functional bearing-area evaluation and multi-criteria optimisation. In conclusion, the integration of cryogenic cooling, 3D functional metrology and advanced optimisation provides a powerful tool for enhancing machining performance and establishing new process standards for shape-memory alloys. The results obtained provide a robust foundation for future research endeavours encompassing intelligent optimisation, adaptive control, and AI-based prediction of surface characteristics in NiTi machining.

The implementation of the TOPSIS method facilitated a comprehensive interpretation of all ten surface parameters, with a clear prioritisation of their functional significance. This finding serves to reinforce the prevailing efficacy of cryogenic cooling techniques, as it has been demonstrated that all of the highest-ranked machining trials were conducted under LN_2_ conditions.

From an industrial perspective, the findings demonstrate unequivocally that cryogenic machining with liquid nitrogen (LN_2_) offers the most reliable and robust pathway to obtaining high-quality NiTi surfaces with low defect density and superior load-bearing characteristics. This has direct implications for the production of medical implants, aerospace components, and precision actuators, where surface integrity critically influences fatigue life, corrosion resistance, and long-term functional stability.

## Figures and Tables

**Table 1:** The chemical composition of NiTi A_f_ = 60 °C—the EDS (Energy-Dispersive Spectroscopy) analysis [25].

Element	wt. [%]	at. [%]
Ti	41.99	47.01
Ni	58.01	52.99
Total	100.00	100.00

**Table 2:** The physical, thermal and mechanical properties of β—NiTi A_f_ = 60 °C [25].

Hardness	Thermal Conductivity	Tensile Strength, Ultimate	Tensile Strength, Yield	Modulus of Elasticity	Density	Structure (Phase)
[HV.1]	[W/m·°C]	[MPa]	[MPa]	[GPa]	[kg/m^3^]	
242	18	1364	649	28	6500	hi-temp B2

**Table 3:** Values of the cutting parameters $f_{z},$ and $v_{c}$ for NiTi alloy, selected by means of the Taguchi method process.

No.	$v_{c}$ [m/min]	$f_{z}$ [mm/tooth]	Cooling Strategy		
1	35	0.02	Dry	MQL	LN_2_
2	35	0.04	Dry	MQL	LN_2_
3	35	0.06	Dry	MQL	LN_2_
4	55	0.02	Dry	MQL	LN_2_
5	55	0.04	Dry	MQL	LN_2_
6	55	0.06	Dry	MQL	LN_2_
7	75	0.02	Dry	MQL	LN_2_
8	75	0.04	Dry	MQL	LN_2_
9	75	0.06	Dry	MQL	LN_2_

**Table 4:** 3D surface texture parameters according to ISO 25178.

No.	$v_{c}$ [m/min]	$f_{z}$ [mm/tooth]	Cooling Strategy	*Sa* [μm]	*Sz* [μm]	*Sq* [μm]	*Sp* [μm]	*Sv* [μm]	*Ssk* [-]	*Sku* [-]	*Smr* [%]	*Smc* [μm]	*Sxp* [μm]
1	35	0.02	LN_2_	0.40	3.67	0.50	1.49	2.18	−0.63	3.31	15.80	0.90	1.19
2	35	0.04	LN_2_	0.36	3.72	0.45	1.72	1.99	−0.31	3.09	4.36	1.16	0.95
3	35	0.06	LN_2_	0.34	3.52	0.42	1.63	1.89	−0.29	3.10	5.48	1.11	0.88
4	55	0.02	LN_2_	0.33	4.11	0.42	2.00	2.11	−0.13	3.52	0.70	1.47	0.85
5	55	0.04	LN_2_	0.38	3.57	0.48	1.42	2.15	−0.44	3.21	19.00	0.83	1.11
6	55	0.06	LN_2_	0.36	3.99	0.45	1.71	2.28	−0.26	3.29	5.00	1.14	0.96
7	75	0.02	LN_2_	0.31	2.99	0.38	1.31	1.68	−0.01	2.86	21.50	0.83	0.75
8	75	0.04	LN_2_	0.37	3.73	0.46	1.52	2.21	−0.21	3.10	13.70	0.92	0.95
9	75	0.06	LN_2_	0.32	2.86	0.39	1.50	1.36	−0.02	2.72	11.40	0.97	0.76
10	35	0.02	Dry	0.43	4.89	0.58	2.29	2.60	−0.30	5.09	2.04	1.59	1.18
11	35	0.04	Dry	0.43	4.94	0.57	2.58	2.37	0.03	4.25	0.82	1.89	1.13
12	35	0.06	Dry	0.54	5.92	0.72	3.18	2.74	0.61	4.14	0.90	2.29	1.22
13	55	0.02	Dry	0.43	5.98	0.56	3.10	2.88	0.02	4.83	0.13	2.43	0.97
14	55	0.04	Dry	1.90	17.40	2.36	5.37	12.00	−0.87	4.28	0.31	2.64	5.60
15	55	0.06	Dry	1.67	24.30	2.35	14.10	10.10	1.02	7.59	0.05	11.50	3.95
16	75	0.02	Dry	0.80	7.18	1.02	4.14	3.04	−0.05	3.04	0.17	2.89	2.10
17	75	0.04	Dry	0.50	6.20	0.65	3.93	2.27	0.44	3.84	0.07	3.09	1.13
18	75	0.06	Dry	0.47	8.30	0.65	3.19	5.11	−1.31	9.61	0.04	2.44	1.40
19	35	0.02	MQL	1.91	17.40	2.33	5.32	12.10	−0.47	3.03	0.64	2.41	4.77
20	35	0.04	MQL	1.16	10.80	1.55	5.23	5.53	−0.09	3.43	0.08	3.04	3.49
21	35	0.06	MQL	1.34	8.75	1.68	4.55	4.20	0.11	2.58	1.46	2.13	3.14
22	55	0.02	MQL	1.95	14.10	2.39	8.63	5.47	0.32	2.71	0.10	5.38	3.94
23	55	0.04	MQL	0.52	5.03	0.67	2.46	2.56	0.17	3.48	2.19	1.62	1.24
24	55	0.06	MQL	0.50	5.50	0.64	2.78	2.71	0.16	3.56	0.64	1.95	1.20
25	75	0.02	MQL	0.88	8.40	1.10	4.14	4.27	0.20	3.08	0.50	2.71	2.01
26	75	0.04	MQL	2.50	30.70	3.22	18.60	12.10	0.66	4.93	0.05	14.80	5.46
27	75	0.06	MQL	0.88	9.62	1.15	4.55	5.07	−0.16	3.74	0.12	3.09	2.41

**Table 5:** The view of the surface after milling for different tests (the isometric images and the Abbott curves).

Test 7: *v_c_* = 75 m/min; *f_z_* = 0.02 mm/tooth; LN_2_		
*Sa* = 0.31 μm *Sz* = 2.99 μm *Sq* = 0.38 μm *Sp* = 1.31 μm *Sv* = 1.68 μm *Ssk* = −0.01 *Sku* = 2.86 *Smr* = 21.50% *Smc* = 0.83 μm *Ssp* = 0.75 μm	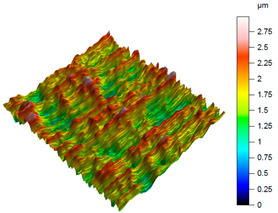	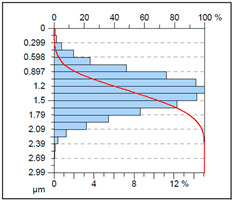
Test 16: *v_c_* = 55 m/min; *f_z_* = 0.06 mm/tooth; Dry		
*Sa* = 1.67 μm *Sz* = 24.30 μm *Sq* = 2.35 μm *Sp* = 14.10 μm *Sv* = 10.1 μm *Ssk* = 1.02 *Sku* = 7.59 *Smr* = 0.05% *Smc* = 11.5 μm *Ssp* = 3.95 μm	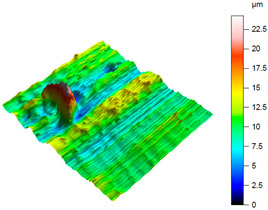	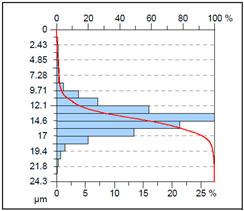
Test 26: *v_c_* = 75 m/min; *f_z_* = 0.04 mm/tooth; MQL		
*Sa* = 2.5 μm *Sz* = 30.7 μm *Sq* = 3.22 μm *Sp* = 18.16 μm *Sv* = 12.1 μm *Ssk* = 0.66 *Sku* = 4.93 *Smr* = 0.05% *Smc* = 14.8 μm *Ssp* = 5.46 μm	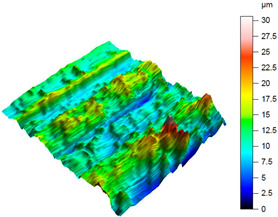	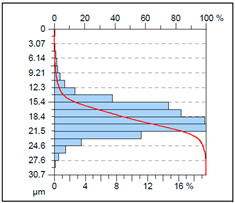

**Table 6:** Final ranking of machining conditions based on the multi-criteria TOPSIS analysis (Weights: 50% Amplitude, 20% Distribution, 30% Functional).

Test No.	$v_{c}$ [m/min]	$f_{z}$	Cooling Strategy	TOPSIS_Score (*C*i)	Rank
7	75	0.02	LN_2_	0.40	1
9	75	0.06	LN_2_	0.36	2
1	35	0.02	LN_2_	0.34	3
8	75	0.04	LN_2_	0.33	4
4	55	0.02	LN_2_	0.38	5
3	35	0.06	LN_2_	0.36	6
2	35	0.04	LN_2_	0.31	7
6	55	0.06	LN_2_	0.37	8
5	55	0.04	LN_2_	0.32	9
17	75	0.04	Dry	0.43	10
16	75	0.02	Dry	0.43	11
24	55	0.06	MQL	0.54	12
12	35	0.06	Dry	0.43	13
13	55	0.02	Dry	1.90	14
23	55	0.04	MQL	1.67	15
10	35	0.02	Dry	0.80	16
27	75	0.06	MQL	0.50	17
11	35	0.04	Dry	0.47	18
25	75	0.02	MQL	1.91	19
18	75	0.06	Dry	1.16	20
21	35	0.06	MQL	1.34	21
14	55	0.04	Dry	1.95	22
20	35	0.04	MQL	0.52	23
22	55	0.02	MQL	0.50	24
15	55	0.06	Dry	0.88	25
19	35	0.02	MQL	2.50	26
26	75	0.04	MQL	0.88	27

## Data Availability

The original contributions presented in this study are included in the article. Further inquiries can be directed to the corresponding author.
